# Evaluation of Potential Probiotic Properties of a Strain of *Lactobacillus plantarum* for Shrimp Farming: From Beneficial Functions to Safety Assessment

**DOI:** 10.3389/fmicb.2022.854131

**Published:** 2022-03-24

**Authors:** Cong Wei, Kai Luo, Mingyang Wang, Yongmei Li, Miaojun Pan, Yumeng Xie, Guangcai Qin, Yijun Liu, Li Li, Qingbing Liu, Xiangli Tian

**Affiliations:** ^1^The Key Laboratory of Mariculture, Ocean University of China, Ministry of Education, Qingdao, China; ^2^Function Laboratory for Marine Fisheries Science and Food Production Processes, Qingdao National Laboratory for Marine Science and Technology, Qingdao, China; ^3^Qingdao Ruizi Group Co., Qingdao, China

**Keywords:** probiotic safety, virulence, antibiotic resistance, genomic analysis, probiotics, *Lactobacillus plantarum*, *Penaeus vannamei*

## Abstract

In recent years the safety of probiotics has received increasing attention due to the possible transfer and spread of virulence factors (VFs) and antibiotic resistance genes (ARGs) among microorganisms. The safety of a strain of *Lactobacillus plantarum* named W2 was evaluated in phenotype and genotype in the present study. Its probiotic properties were also evaluated both *in vivo* and *in vitro*, including adherence properties, antibacterial properties and beneficial effects on the growth and immunity of Pacific white shrimp, *Penaeus vannamei*. Hemolysis tests, antibiotic resistance tests and whole genome sequence analysis showed that W2 had no significant virulence effects and did not carry high virulence factors. W2 was found to be sensitive to chloramphenicol, clindamycin, gentamicin, kanamycin and tetracycline, and to be resistant to ampicillin and erythromycin. Most ARGs have no transfer risk and a few have transfer risk but no significant enrichment in human-associated environments. The autoaggregation of W2 was 82.6% and the hydrophobicity was 81.0%. Coaggregation rate with *Vibrio parahaemolyticus* (24.9%) was significantly higher than *Vibrio*’s autoaggregation rate (17.8%). This suggested that W2 had adhesion potential to mucosal/intestinal surfaces and was able to attenuate the adherence of *V. parahaemolyticus*. In addition, several adhesion-related protein genes, including 1 S-layer protein, 1 collagen-binding protein and 9 mucus-binding proteins were identified in the W2 genome. W2 had efficiently antagonistic activity against 7 aquatic pathogenic strains. Antagonistic components analysis indicated that active antibacterial substances might be organic acids. W2 can significantly promote the growth of shrimp when supplemented with 1 × 10^10^ cfu/kg live cells. Levels of 7 serological immune indicators and expression levels of 12 hepatopancreatic immune-related genes were up-regulated, and the mortality of shrimp exposed to *V. parahaemolyticus* was significantly reduced. Based on the above, *L. plantarum* W2 can be applied safely as a potential probiotic to enhance the growth performance, immunity capacity and disease resistance of *P. vannamei*.

## Introduction

*Lactobacillus plantarum* is a kind of highly adaptable lactobacillus that is widely found in various fermented products of plants and animals and occurs naturally in the intestinal tract of animals including humans (Xu et al., 2015). Some strains have already been used as probiotics and play a crucial role in human health, livestock rearing and aquaculture. For example, *L. plantarum* 0612 competes for surface receptors on human intestinal Caco-2 epithelial cells and significantly inhibits the adhesion of *Escherichia coli* and *Listeriosis monocytogenes* (Lau and Chye, 2018). *L. plantarum* CAM6 can be used as an antibiotic alternative for weaned pigs (Betancur et al., 2020). *L. plantarum* is generally used as an effective probiotic in aquaculture, and has been widely studied and applied in shrimp and fish culture. Several strains of *L. plantarum* have been shown to enhance resistance to *V. alginolyticus* (Chiu et al., 2007), *V. harveyi* (Vieira et al., 2010; Kongnum and Hongpattarakere, 2012), *V. parahaemolyticus* (Thammasorn et al., 2017) infections in shrimp. *L. plantarum* FNCC 226 can alleviate parasitism and growth inhibition of *Pangasius hypophthalamus* by *Saprolegnia parasitica* (Nurhajati et al., 2012). Several strains have been found to have beneficial effects on the growth, immunity and disease resistance in various fish species, such as *Anguilla japonicav* (Lee et al., 2017), *Oncorhynchus mykiss* (Vendrell et al., 2008), *Sparus aurata* (Picchietti et al., 2007), *Labeo rohita* (Giri et al., 2013), *Oreochromis niloticus* (Van Doan et al., 2017), and *Paralichthys olivaceus* (Beck et al., 2015).

Safety assessment is a vitally important task for the screening of probiotics to exclude adverse effects (FAO/WHO, 2002). Accurate identification, adequate phenotypic characterization and assessment of potential risks are indispensable steps in the safety assessment of probiotics (Miquel et al., 2015; Reid et al., 2019). Generally, *Lactobacillus* can be used safely in most cases. For example, there are only sporadic cases of *Lactobacillus* acting as a clinical infection pathogen in humans, with a very small proportion of *L. plantarum* involved (Haghighat and Crum-Cianflone, 2016; Nayeem et al., 2018; Koyama et al., 2019). Bernardeau et al. (2006) estimated that the risk of *Lactobacillus* infection was extremely low, with approximately one case per 10 million people over a period of more than a century in France.

Until now, no cases of animal infection have been reported. However, safety of probiotics has received increasing attention in recent years due to the potential transfer and spread of antibiotic resistance genes (ARGs) among microorganisms (Wang et al., 2008; Doron and Snydman, 2015). Previous studies have shown that the extensive use of antibiotics not only enhances antibiotic resistance in bacteria, but also promotes the transfer of ARGs (Santajit and Indrawattana, 2016). A probiotic should have a low risk of transferring ARG to the environment. However, an antibiotic resistance profile of *Lactobacillus* demonstrated that acquired resistance genes were occasionally present in *Lactobacillus* species (Campedelli et al., 2019).

*L. plantarum* is included in the Qualified Presumption of Safety (QPS) list of European Food Safety Authority (EFSA, 2008). When supplementing with live cells, it is recommended to assess the safety of the strain in use by QPS. EFSA guidance specifies four main aspects of bacterial safety assessment: strain identification; strain toxicity and pathogenicity; antibiotic resistance; and antimicrobial substance identification (EFSA, 2018). It is clear in the guidance that virulence factors (VFs) should be excluded in *Enterococcus faecalis* (IS16, hylEfm, esp) and *Bacillus* (enterotoxin, cereulide synthase), while those in *L. plantarum* are not listed. In response to the transfer of ARGs, EFSA delineated the cut-off value of *L. plantarum* to 7 antibiotics.

In terms of biological and ecological safety, probiotic screening should be carried out through a careful, phenotypically and genotypically based strategy according to the EFSA guidance (EFSA, 2018). In addition, it is essential to consider the beneficial effects of a probiotic candidate in both laboratory and practical applications, and to uncover the related mechanisms. Unfortunately, due to limited research and knowledge, there are still no internationally accepted screening criteria for probiotics in aquaculture which balance safety and beneficial effects. Therefore, it is important to develop standardized evaluation criteria to screen probiotics for application in aquaculture.

A strain of *Lactobacillus* was isolated from the intestine of Pacific white shrimp, *Penaeus vannamei* in our laboratory and named W2. In accordance with the latest EFSA guidelines, this study investigates the safety of the W2 strain in genotype and phenotype, including the assessment of virulence and VFs, antibiotic resistance and ARGs, the origin of VFs and ARGs, and the possible transfer potential of acquired genes. The potential probiotic properties of the strain for shrimp farming are also investigated, including the potential for adhesion, antagonistic activity *in vitro* and any modulatory effects on shrimp growth, immunity and disease resistance when supplemented in feed. The results from this study would be helpful to develop criteria for the screening and evaluation of probiotics in aquaculture.

## Materials and Methods

### Bacterial Strain Identification

The strain of *L. plantarum* named W2 was isolated from the intestinal tract of Pacific white shrimp, *P. vannamei*, and was obtained from the Microbial Culture Centre, Laboratory of Aquaculture Ecology, Ocean University of China, China. W2 was identified using physiological and biochemical assays and 16S rRNA sequencing. The 16S rRNA was amplified with primers 27F (5′-AGAGTTTGATCCTGGCTCAG-3′) and 1492R (5′-GGTTACCTTGTTACGACTT-3′). 16S rRNA sequences were available through Sanger. 19 strains were selected in the NCBI database to construct a phylogenetic tree (MEGAX, Neighbor-Joining).

### Assessment of Potential Probiotic Properties

#### Adhesion Assay *in vitro*

The microbial adhesion to solvents (MATS) method was employed to determine the hydrophobicity and electron donor-acceptor properties of W2 (Bellon-Fontaine et al., 1996). The autoaggregation and coaggregation assays of bacteria were as described by Kos et al. (2003) with some modifications. Hydrophobicity, autoaggregation, coaggregation were calculated using the following formulas:


$$\mathrm{Hydrophobicity}\left(\%\right)=\left[\left(A_{x}-A_{t}\right)/A_{x}\right]\times 100\%$$

$$\text{Auto}\mathrm{aggregation}\left(\%\right)=\left(1-A_{p}\right)/A_{x}\times 100\%$$

$$\mathrm{Coaggregation}\left(\%\right)=\left[\left(A_{x}+A_{y}\right)/2-A_{\left(x+y\right)}\right]/\left(A_{x}+A_{y}\right)\times 100\%$$

W2 was cultured in MRS broth at 37°C for 20 h and pathogens (*V. splendens*, *V. parahaemolyticus*, *V. vulnificus*) were cultured in broth medium at 26°C for 24 h, then, centrifuged at 5,000 g for 10 min and the supernatant discarded. The bacteria were diluted with sterile saline until reaching an absorbance value of around 0.8 (OD 600), recorded as *A*_*x*_ and *A*_*y*_. The W2 suspension was fully mixed 3:1 with hydrophobic agent (n-dodecane, xylene, chloroform), allowed to settle for 20 min at 25°C and the OD600 of the aqueous phase was measured and recorded as *A*_*t*_. Four ml of bacterial suspension was allowed to stand for 5 h at 25°C and the OD600 of the surface suspension measured and recorded as *A*_*p*_. The W2 suspension was fully mixed 1:1 with the pathogens, allowed to settle for 5 h at 25°C, and the OD600 of the surface suspension measured and recorded as *A_(x+y)_*. Each treatment consisted of three replicates and was repeated three times.

#### Antagonistic Assay *in vitro*

The antagonistic activity of W2 was evaluated using the agar diffusion method (Oxford cup method) (Kuang et al., 2018). The selected indicator bacteria were 7 common aquatic pathogenic strains, including *V. vulnificus* S01P2, *V. splendidus* BSD11, *V. harveyi* SRTT9 (Wang et al., 2020), *V. parahaemolyticus* 20130629002S01 (Dong et al., 2017), *Streptococcus iniae* NUF849 (Sheng et al., 2018), *Aeromonas hydrophila* AP40301 (Xu et al., 2018), and *Shewanella marisflavi* AP629 (Li et al., 2010). The culture methods of W2 and pathogens were similar to those described in the adhesion assay. Pathogenic bacteria fermentation broth was centrifuged at 5,000 *g* for 10 min and the supernatant discarded. It was then diluted with broth medium to a final concentration of 1 × 10^6^ cfu/ml. Different pathogen suspensions were coated onto agar plates and dried for 15 min. An Oxford cup was vertically placed on the plate, and then 200 μl of W2 suspension was dispensed into the cup and allowed to diffuse at 26°C for 24 h. A control experiment was conducted using MRS broth. The inhibition zone diameters were then measured.

For screening for antibacterial activity, the culture broth (CB), cell free supernatant (CFS), and biomass suspension (BS) were used in an antagonistic assay, with *V. vulnificus* S01P2 and *V. parahaemolyticus* 20130629002S01 as the indicator bacteria. The CFS was further processed as follows: 1 g/ml Tripsin was added to deproteinated supernatant (CFT). The supernatant was heated to 80°C for 10 min to remove H_2_O_2_ (CFH), then the pH was adjusted to 6.0 to exclude the antimicrobial effect of organic acids (CF6.0). Each treatment consisted of three replicates and was repeated three times.

### Safety Assessment

#### Hemolytic Activity

The hemolytic activity was tested using the Oxford cup diffusion test (Kuang et al., 2018). Colombian blood plate (5% sheep blood) was purchased from ELITE-MEDIA. MRS broth was used as the negative control. W2 was incubated in MRS broth for 24 h and the supernatant was obtained by centrifugation. The pH of the supernatant and broth was adjusted to 6.0 to exclude the effects of acidity. The Oxford cups were placed on the blood plate and filled with 200 μl supernatant or broth. The plate was incubated at 28°C for 24 h, then observed for the hemolytic type. Each consisted of three replicates and was repeated three times.

#### Antibiotic Resistance

Based on the EFSA (2018) guidelines, this study determined the minimum inhibitory concentration (MIC) of 7 antibiotics (ampicillin, kanamycin, chloramphenicol, clindamycin, erythromycin, gentamicin, and tetracycline) against W2 using the micro-broth dilution method (El Shazely et al., 2020). *Escherichia coli* ATCC 25,922 was used as the standard strain. Separate solutions of 512 μg/ml of the 7 antibiotics mentioned above were prepared. The concentrations of antibiotics in wells 1–11 of the 96-well plate were 128, 64, 32, 16, 8, 4, 2, 1, 0.5, 0.25, 0.125, and 0.0625 μg/ml, respectively. 100 μl bacterial suspension (OD600: 0.1) was added to each well and incubated for 20 h in 37°C. The results were then observed. Each treatment consisted of three replicates and was repeated three times.

#### Whole Genome Sequencing and Analysis

Whole genome sequencing (WGS) was performed on the Illumina Hiseq and PacBio platforms. Sequencing quality control was done with Fastp (Chen et al., 2018), using an average quality score of 20 and a minimum read length threshold of 30 bp. Assembly was performed using Unicycler v0.4.8 (Wick et al., 2017), with parameters that mainly included kmer values ranging from 21 to 41. The softwares used for gene annotation were DIAMOND v0.8.22 (Buchfink et al., 2015) for NR, KOG, Swiss-Prot, and KEGG (*e*-value = 1e-5); blast2GO (Conesa and Götz, 2008) for GO (*e*-value = 1e-5); HMMER 3.0 (Eddy, 2009) for Pfam (*e*-value = 0.01). ARGs and VFs were obtained by comparison in the CARD (The Comprehensive Antibiotic Research Database) and the VFDB (Virulence Factor Database). Mobile genetic elements (MGEs) were analyzed in W2. Genomic island (GEIs) prediction was performed using IslandViewer (Bertelli et al., 2017), pre-phages were predicted using Phage_Finder (Fouts, 2006), CRISPR-Cas was predicted using MinCED (Bland et al., 2007), integrons in DNA sequences were detected using Integron_Finder (Cury et al., 2016), and insertion sequence (IS) elements in the genome were identified using ISEScan (Siguier et al., 2014). The sequencing data were deposited in the Sequence Read Archive (SRA) repository under the accession number PRJNA797524.

### Feeding Trial and Challenge Test for Shrimp

#### Shrimp Feeding Trial and Sample Collection

To determine the probiotic effects of W2 on aquaculture animals, a 42-day feeding trial of *P. vannamei* was designed. 225 healthy juvenile shrimps were randomly divided into three treatments. Five replicates were established for each treatment. The three treatments were as follows: (1) shrimp fed basal diet (the control, CON); (2) shrimp fed basal diet supplemented with 1 × 10^10^cfu/kg live cells (LLP); (3) shrimp fed basal diet supplemented with 15 mg/kg florfenicol for 7 d in a 14-day interval (FLI). Florfenicol treatment was set up as a positive control to compare with the effects of W2 in shrimp. Nutritional compositions of the basal diet are given in Supplementary Table 1. During the experiment, the water was exchanged one third of the aquarium volume (53 × 28 × 34 cm, 50 L) and the shrimp were fed four times per day (08:00, 12:00, 16:00, and 20:00).

Uneaten feed was collected 1 h after feeding, dried at 60°C and weighed. At the end of the feeding trial, the shrimp were starved for 24 h. Then the shrimp were counted, weighed and their tissues were collected. The hemolymph of the shrimp was collected without anticoagulants and stored at 4°C. After 24 h, serum was obtained by centrifugation at 3,000 *g* for 10 min. Hepatopancreas was collected and immediately frozen in liquid nitrogen. Both serum and hepatopancreas were stored at −80°C waiting for determination of the activities of non-specific immune enzymes activity and expression levels of immune-related genes.

#### Immunological Parameters Determination

Based on the innate immune properties of crustaceans, the following 8 serological indicators of immune function of shrimp were measured: Alkaline phosphatase (AKP), acid phosphatase (ACP), total nitric oxide synthase (T-NOS), peroxidase (POD), superoxide dismutase (SOD), phenol oxidase (PO) activities, total antioxidant capacity (T-AOC), and lysozyme (LZM) content. They were quantified using commercial detection kits obtained from the Nanjing Jiancheng Bioengineering Institute (Nanjing, China). The relative expression levels of the following 12 immune genes in the hepatopancreas of the shrimp were determined: *Imd*, *Toll*, *Relish*, *TOR*, *4E-BP*, *eIF4E1α*, *eIF4E2*, *SOD*, *LZM*, *proPO*, *HSP70*, and *LGBP*. All the above immune-related genes are showed in Supplementary Table 2, with gene description and primers used. Total RNA was extracted using Trizol Reagent (Takara, Japan). The quantity and purity of RNA samples were examined using a NanoDrop spectrophotometer (Thermo Fisher Scientific, United States), followed by 1.5% agarose gel electrophoresis. Total RNA was reverse-transcribed to cDNA using the PrimeScript^^TM^ RT reagent Kit (Takara, Japan). Real-time quantitative RT-qPCR was performed using the QuantStudio^^TM^ 5 real-time PCR system (Applied Biosystems, United States).

#### Challenge Test

After the feeding trial, the remaining shrimp were fed continually for 5 additional days. 24 shrimp were randomly selected from each treatment and divided into 3 replicates for the challenge experiment. *Vibrio parahaemolyticus* 20130629002S01 were provided by the Microbial Culture Centre, Laboratory of Aquaculture Ecology, Ocean University of China, China. The LC_50_ of *V. parahaemolyticus* on 14th day was 5 × 10^7^ cfu/ml determined during the pre-experiment. Shrimp were injected intramuscularly in the third abdominal segment with live *V. parahaemolyticus* at the concentration of 5 × 10^7^ cfu/ml. Daily management during the challenge test was the same as that during the feeding trial. Cumulative mortality of shrimp was calculated on the 14th day after injection.

### Calculations and Statistical Analysis

The growth performance of shrimp is described by the survival rate (SR, %), specific growth rate (SGR, %/d) and feed efficiency rate (FER). These formulas are defined as follows:


$$\text{SR}=N_{t}\times 100/N_{0}$$

$$\text{SGR}=\left(\ln\,W_{t}-\text{ln}\,W_{0}\right)\times 100/t$$

$$\text{FER}=\left(W_{t}-W_{0}\right)\times 100/F_{0}$$

where *N*_0_ and *N*_*t*_ represent the number of shrimp at the beginning and end of the culture, respectively; *W*_0_ and *W*_*t*_ represent the initial and final weights of the shrimp (g); *t* refers to the duration of the experiment (d); and *F*_0_ refers to the feed intake (dry weight, g).

All non-genomic data in this study were subjected to one-way ANOVA (SPSS 22.0). Data were tested for normality, homogeneity and independence prior to ANOVA. Results are represented as mean ± standard error.

## Results

### Strain Identification

**FIGURE 1:**
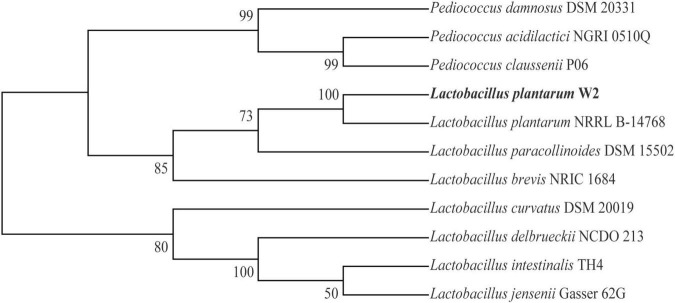
Phylogenetic tree of W2 based on 16S rRNA.

The physiological and biochemical properties of W2 are presented in Supplementary Table 3. W2 is a Gram-positive *bacillus*, non-budding, non-motile, catalase negative, and produces acid but not acetyl methyl methanol and not hydrogen sulfide. W2 can ferment most monosaccharides and oligosaccharides except xylose. The identification was consistent with the basic characteristics of *L. plantarum*. A phylogenetic tree constructed based on 16S rRNA sequences confirmed that W2 is a strain of *L. plantarum* (Figure 1).

### Adhesion Properties *in vitro*

**FIGURE 2:**
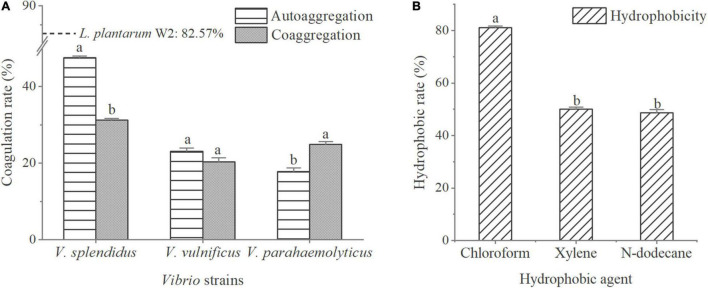
Adhesion properties of *L. plantarum* W2. **(A)** Autoaggregation rates of three *Vibrio* strains (*V. splendidus* BSD11, *V. vulnificus* S01P2, *V. parahaemolyticus* 20130629002S01) and their coaggregation rate with W2. Autoaggregation rate of W2 is indicated by a dotted line. **(B)** W2 adherence to hydrophobic agent (chloroform, xylene, n-dodecane). *P* < 0.05.

The adhesion properties of W2 are shown in Figure 2. The autoaggregation rate of W2 was 82.6% compared to less than 50.0% for the three *Vibrio* strains. The coaggregation rates of W2 with the three *Vibrio* strains were 31.2% for *V. splendidus*, 20.4% for *V. vulnificu*, and 24.9% for *V. parahaemolyticus*. This indicated that the interaction between W2 and *Vibrio* significantly enhanced the aggregation of *V. parahaemolyticus* (*P* < 0.05) and significantly reduced the aggregation of *V. splendens* but had no significant effect on the aggregation of *V. vulnificu* (*P* > 0.05). In the hydrophobicity assays, W2 showed the highest hydrophobicity in chloroform (81.0%), which was 66.5 and 61.8% higher than n-dodecane and xylene, respectively. Thus, W2 has a high autoaggregation rate (82.6%), a high hydrophobicity (chloroform: 81.0%), and it is strain-specific in co-coaggregation with different *Vibrio* strains. It can significantly reduce the adhesion of *V. parahaemolyticus*.

The extracellular protein/transmembrane protein genes associated with adhesion in W2 are presented in Table 1. 1 S-layer protein, 1 collagen binding protein (CnBP) and 9 mucus binding proteins (Mub) were identified in W2 based on Pfam and NR database annotations.

**TABLE 1:** Adhesion-associated proteins in W2 genome.

Protein name	Numbers	Genes location
Mucus binding proteins (Mub)	9	Gene0330, gene1041, gene2166, gene1016, gene1387, gene0768, gene2725, gene2666, gene2713
Collagen binding protein (CnBP)	1	Gene2581
S-layer protein	1	Gene3124

### Antagonistic Activity *in vitro*

**FIGURE 3:**
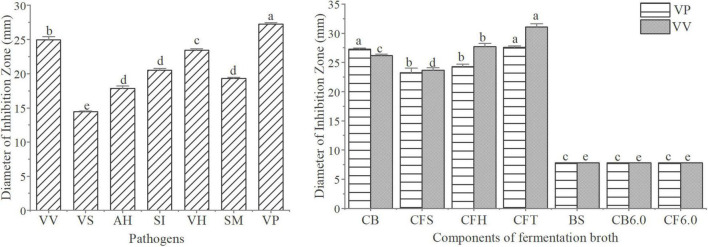
Analysis of the antagonistic activity of W2. **(A)** Antibacterial activitys of W2 against seven pathogen (*V. vulnificus* S01P2, *V. splendidus* BSD11, *V. harveyi* SRTT9, *V. parahaemolyticus* 20130629002S01, *Streptococcus iniae* NUF849, *Aeromonas hydrophila* AP40301, *Shewanella marisflavi* AP629). **(B)** Antibacterial activitys of different fermentation components of W2 against two Vibrios (*V. parahaemolyticus* 20130629002S01, *V. vulnificus* S01P2). The groups are as follows: culture broth (CB), cell free supernatant (CFS), cell free supernatant with H_2_O_2_ removal (CFH), cell free supernatant treated with tripsin (CFT), biomass suspension (BS), cell free supernatant at pH 6.0 (CB6.0), culture broth at pH 6.0 (CF6.0). *P* < 0.05.

The antagonistic activity of W2 to seven aquatic pathogenic strains is shown in Figure 3A. The average inhibition zone diameter of W2 against pathogens (*V. vulnificus* S01P2, *V. harveyi* SRTT9, *V. parahaemolyticus* 20130629002S01, *Streptococcus iniae* NUF849) was > 20 mm. The diameters of the inhibition zones of different fermentation components of W2 against 2 *Vibrio* strains (*V. parahaemolyticus* 20130629002S01 and *V. vulnificus* S01P2) are shown in Figure 3B. The diameter of the inhibition zone of the suspension was 7.8 mm, which is the diameter of the Oxford cup and is considered as indicating no antagonistic activity. The zone diameters of the Fermentation broth and supernatant (*V. parahaemolyticus*: 26.2, 23.7; *V. vulnficus*: 27.3, 23.3) were significantly larger than 7.8 mm (*P* < 0.05), indicating that the antagonistic active substance existed in the supernatant. The diameter of the inhibition zones of the fermentation broth was larger than that of the supernatant, probably due to the continued production of antagonistic substances during the experiment. When the pH was adjusted to 6.0 in the fermentation broth and supernatant, their antagonistic activity disappeared. The inhibition zone diameter of the supernatant did not decrease after the removal of H_2_O_2_ and treatment with trypsin.

### Antibiotic Resistance

MICs of seven antibiotics against W2 are presented in Table 2. The cut-off value represents the maximum value of MIC assigned by EFSA, which specifies the maximum resistance level the bacteria group should meet. The resistance of W2 to chloramphenicol (8 μg/ml), clindamycin (4 μg/ml), gentamicin (16 μg/ml) were in accordance with the cut-off values, the resistance to kanamycin (16 μg/ml), tetracycline (2 μg/ml) were below the cut-off values, while the resistance to ampicillin (8 μg/ml) and erythromycin (8 μg/ml) were above the cut-off values. This means that W2 can be inhibited in growth by lower concentrations of kanamycin and tetracycline than the cut-off values, but is resistant to ampicillin and erythromycin at cut-off value concentrations.

**TABLE 2:** Minimum inhibitory concentration (MIC) of *L. plantarum* W2.

Antibiotics	MIC (μg⋅ml^–1^)	
	Cut-off value	Test values
Ampicillin	2	8 (R)
Kanamycin	64	16 (S)
Chloramphenicol	8	8 (S)
Clindamycin	4	4 (S)
Erythromycin	1	8 (R)
Gentamicin	16	16 (S)
Tetracycline	32	2 (S)

*S means L. plantarum W2 was sensitive to the antibiotic, and R means resistance to the antibiotic.*

By alignment with the CARD database, 155 ARGs were annotated in the W2 WGS. Their detailed distributions in different antibiotic classes are presented in Supplementary Table 4. ARGs in W2 were mainly concentrated in macrolide, tetracycline, fluoroquinolone, phenicol, lincosamide, peptides, penam, streptogramin, and oxazolidinone antibiotic. Based on the MIC results, erythromycin and ampicillin resistance genes were analyzed in this study, and the results are presented in Supplementary Table 5. 50 macrolide resistance genes were identified and they were classified by resistance mechanism into 42 antibiotic effluxs, 7 antibiotic target protection, and 1 antibiotic target alteration. The antibiotic efflux includes 3 *efrA*, 2 *evgA*, 25 *macB*, 1 *MexL*, 3 *mtrA*, 5 *oleC*, 3 *Staphylococcus aureus LmrS*. Antibiotic target protection includes l *mrC*, 1 *lsaA*, 1 *lsaC*, 2 *optrA*, 1 *tva(A)*, 1 *vmlR*. The antibiotic target alteration gene is *Erm(K)*. 11 penam resistance genes were found, grouped into 7 resistance efflux pumps (3 *mtrA*, *mgrA*, 2 *evgA*, *golS*), 1 antibiotic target protection (*mecI*) and 3 antibiotic inactivation (*Escherichia coli ampH beta-lactamase*, *y56 beta-lactamase*, *NmcR*). In addition to the CARD, 5 penicillin-binding proteins (PBPs) were annotated using the Swiss-Prot database, 13 β-lactamases using the pfam database, 1 penicillin V acylase using the NR database and 1 penicillin amidase using the GO database. Some of the above genes may have mediated the additional high resistance of W2 to erythromycin and ampicillin. Vancomycin resistance genes are not present in W2.

### Virulence

**FIGURE 4:**
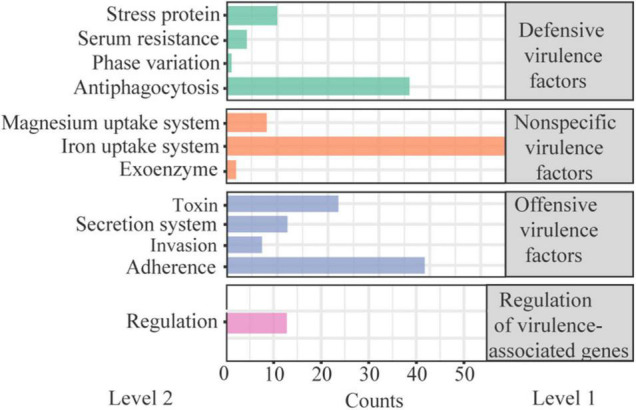
Classification of virulence-related factors (VFs) present in W2 genome.

There were 274 VFs annotated in W2 in the VFDB. Of these, 202 were attribute to specific I/II level, with a few genes in multiple II level functions (Figure 4). Defensive virulence factors, nonspecific virulence factors and regulation of virulence-associated genes accounted for 62.0%, and offensive virulence factors for 38.0%. Toxin accounted for 27.5% (22) of the offensive virulence factors, with the other factors annotated to adhesion, invasion and secretion systems. The 22 toxins included 4 Alpha-hemolysin, 11 Beta-hemolysin/cytolysin, 1 Cytolysin, 1 Colibactin, 1 Hemolysin III, 3 RTX toxins, and 1 TcdA. Their locations on the DNA are presented in Supplementary Table 6. The haemolysis test showed that both the fermentation supernatant of W2 and the MRS broth (negative control) represent γ haemolysis. This suggests that the haemolysin/cytolysin gene of W2 is not expressed, or slightly expressed without haemolytic effect.

### Transfer Potential of Safety-Related Genes

In this study, MGEs in W2 (Table 3) were analyzed to assess the potential for horizontal transfer of ARGs and VFs. The W2 genome has only one circular DNA and no plasmids. The following MGEs were identified on the circular DNA: 6 genomic islands (GEIs), comprising a total of 200 genes; 2 prephages (*Lactococcus* phage P335 and *Lactobacillus* phage phig1e), comprising a total of 99 genes, overlapping with 2 of the 6 genomic islands; 3 insertion sequences (IS); 10 CRISPR sequences (CRISPRs). In addition, 8 possible integrases and 10 possible transposases (3 of which are insertion sequences) were identified by protein sequence comparison in the Swiss-Prot database. No complete integron was found.

**TABLE 3:** Mobile genetic elements (MGEs) and associated ARGs and VFs statistics.

MGEs	Counts	ARGs	VFs
Plasmid	0	0	0
Integron	0	0	0
Prephage	2	3	5
GEIs	6	7	10
CRISPRs	10	0	0
Integrase	8	3	4
Transposase	9	1	2
IS	3	0	0

**FIGURE 5:**
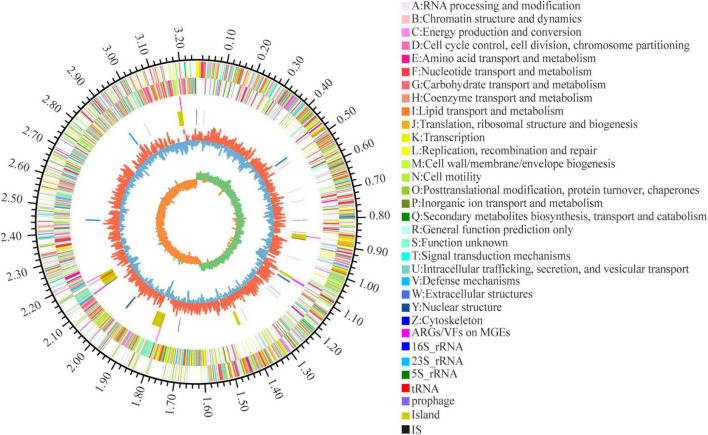
Circular representation of *L. plantarum* W2 genome. The outer scale is in mega bases (Mb). Circle 1 (from outside to inside), the marker of genome size. Cycles 2 and 3, CDS with positive and negative chain, different colors represent different functional classifications; Circle 4, ARGs and VFs located on genomic islands and prephages; Circle 5, islands and prephages (yellow), insertion sequences (IS, black), rRNA (blue) and tRNA (red); Circle 6, GC content, the higher value makes redder, the lower makes bluer. Circle 7, GC-skew value, skew+ are expressed in green, skew- are expressed in orange.

Seven of the 155 ARGs were found located on GEIs. Of these, 4 mediated macrolide resistance (*efrA*, *macB*), 3 mediated fluoroquinolone resistance (*patB*, *efrA*), 2 mediated rifamycin resistance (*efrA*), 1 mediated nitroimidazole resistance (*msbA*), and 1 mediated tetracycline resistance (*tetT*). 10 of the 274 VFs are located on GEIs/pre-phages, with 2 genes encoding toxins (TcdA, RTX toxin). A total of 11 ARGs/VFs were located on GEIs, due to partial overlap between 7 ARGs and 10 VFs. In addition, 6 VFs and 4 ARGs are located near the integrase/transposase. The location of MGEs such as GEIs, prephages, IS, and the ARGs and VFs carried on them are shown in Figure 5 in circle 5, 4.

### Shrimp Growth Performance

The growth performance of shrimp in each treatment is presented in Table 4. The data indicated that the specific growth rate (SGR) of shrimp of LLP and FLI were significantly higher than that of the control (*P* < 0.05). The feed utilization efficiency (FER) was significantly higher in the LLP and FLI than in the control, but there was no significant difference between the two treatments.

**TABLE 4:** Growth performance of *P. vannamei*.

Treatment	Initial weight/g	Final weight/g	SGR%/d	FER%	SR%
CON	0.94 ± 0.01*^a^*	6.01 ± 0.14*^b^*	4.42 ± 0.06*^b^*	73.8 ± 3.48*^b^*	89.33 ± 3.40*^a^*
LLP	0.93 ± 0.02*^a^*	7.10 ± 0.17*^a^*	4.84 ± 0.08*^a^*	85.2 ± 3.56*^a^*	85.33 ± 3.89*^a^*
FLI	0.95 ± 0.01*^a^*	6.79 ± 0.23*^a^*	4.68 ± 0.10*^a^*	86.5 ± 1.90*^a^*	90.67 ± 1.63*^a^*

*Data with different letters indicate significant difference with each other (P < 0.05).*

### Shrimp Immune Parameters

**FIGURE 6:**
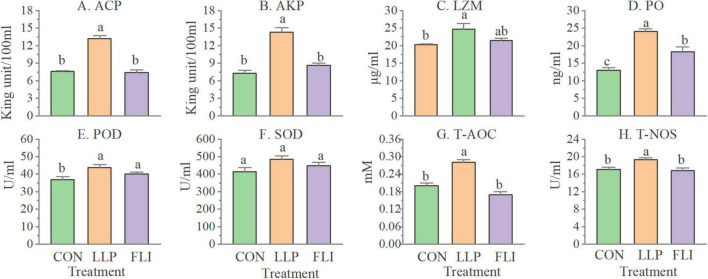
Levels of serological immune indicators of *P. vannamei.*

**FIGURE 7:**
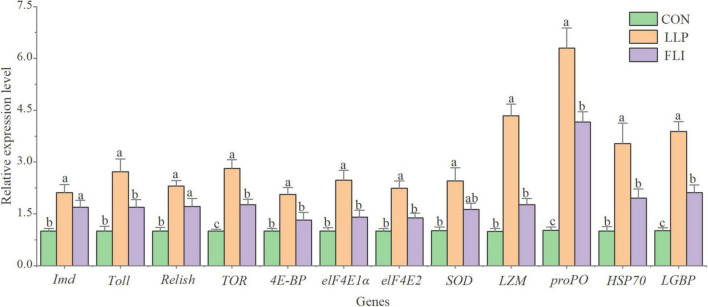
Expression levels of hepatopancreatic immune-related genes of *P. vannamei.*

Levels of 8 serological immune indicators and expression levels of 12 hepatopancreatic immune-related genes of shrimp are shown in Figures 6, 7. AKP, ACP, TNOS, PO, LZM, POD, and T-AOC were significantly higher in the LLP than in the control. PO was significantly higher in the FLI than in the control, but significantly lower than LLP. SOD was not significantly different in the three groups. The relative expression levels of all 12 immune genes were significantly higher in the LLP group than in the control. *Imd*, *Relish*, *TOR*, *proPO*, and *LGBP* were significantly higher in the FLI group than in the control. In summary, levels of serological immune indicators showed the same trend as the relative expression levels of hepatopancreatic immune-related genes. W2 supplementary to the diet significantly improved the immune performance of shrimp (enhanced immune enzyme activity and up-regulation of immune gene expression). Antibiotics florfenicol supplementary to the diet also improved the immune performance of shrimp to some extent, but to a lesser extent than W2.

### Challenge Test

**FIGURE 8:**
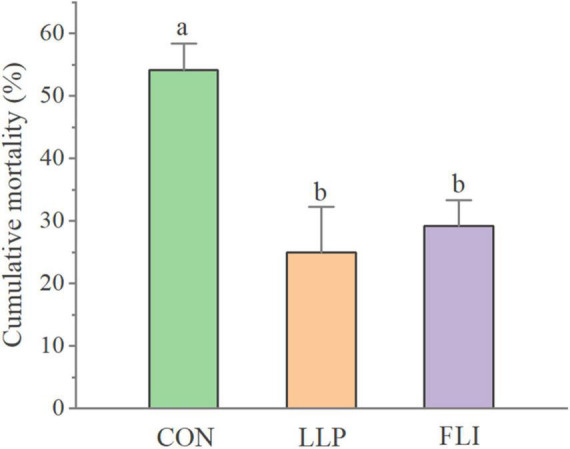
Cumulative mortality of *P. vannamei.*

The cumulative mortality of *P. vannamei* in the challenge test is shown in Figure 8. The LLP group had the lowest cumulative mortality rate (25.0%) and was not significantly different from the FLI group (29.2%). The control group had a high cumulative mortality rate of 54.2%, which was significantly higher than the LLP and FLI (*P* > 0.05). Compared to the control, calculations showed a 53.8 and 46.2% reduction in cumulative mortality in the LLP and FLI groups, respectively.

## Discussion

As one of the eco-friendly alternatives to antibiotics, probiotics have been widely considered and their probiotic effect on aquaculture animals have been confirmed by previous studies (e.g., Li et al., 2019a,b; Tran et al., 2019; Yang et al., 2019). *L. plantarum* was included in the list of microorganisms that can be used in food in many countries and regions such as China, Europe, United States, Australia, and Malaysia (EFSA, 2007; Ministry of Health China, 2010). In this study, the probiotic properties of a *L. plantarum* W2 strain, isolated from the intestine of healthy Pacific white shrimp, were evaluated including the potential for adhesion, antagonistic activity *in vitro* and modulatory effects to growth, immunity and disease resistance of *P. vannamei*.

The adherence of probiotic to epithelial cells is considered to be an important requirement for their colonization and delivery of health effects (Collado et al., 2005; Mohanty et al., 2019). Bacteria develop a cluster through autoaggregation, which facilitates their effective adherence to the intestinal surface (Trunk et al., 2018). Meanwhile, coaggregation between probiotic and pathogen can indicate their intimate interactions (Mohanty et al., 2019). Strain W2 performed high autoaggregation (82.6%) and hydrophobicity (chloroform: 81.0%), which indicated a certain adhesion potential. Notably, its coaggregation with *V. parahaemolyticus* was significantly greater than the autoaggregation of *V. parahaemolyticus*, which might mean W2 could make an intimate connection with *V. parahaemolyticus*, allowing it to release antagonistic substances in a close position (Bujnakova and Kmet, 2002). As a result, W2 might be able to attenuate the adherence and colonization of *V. parahaemolyticus* to some extent.

Adhesion of *Lactobacillus* to mucin/epithelial cells is associated with a variety of non-specific and specific ligand-receptor interactions (Boks et al., 2008; Berne et al., 2018). It is generally agreed that the initial mechanism is linked to physicochemical interactions between bacteria and mucin/epithelial cells. Certain features of bacterial surfaces, such as electron-donor properties, surface charges and hydrophobicity, influence the forces of attraction and repulsion between bacteria and surfaces (Humphries et al., 2017; Ren et al., 2018). Xylene and n-dodecane are non-polar solvents and reflect the hydrophobicity of the bacteria. Chloroform is a monopolar and acidic solvent and it served as an indicator of the electron acceptor feature of the bacteria (Bellon-Fontaine et al., 1996). W2 demonstrated a significantly higher hydrophobicity toward chloroform, which meant it was a strong electron donor. This might play an important role in the early stages of adhesion. Meanwhile, special bacterial surface components, such as extracellular proteins/transmembrane proteins, can strengthen the initial contact and play a major role in adhesion (Bath et al., 2005; Tallon et al., 2007; Berne et al., 2013). Previous studies have revealed a variety of proteins associated with *Lactobacillus* adhesion, including S-layer protein (Mohanty et al., 2019), collagen-binding protein (CnBP) (Roos et al., 1996), mucus-binding protein (Mub) (Roos and Jonsson, 2002), mannose-specific adhesin (Msa) (Pretzer et al., 2005), mucus-binding flagellin (SpaC), and cell wall-anchored protein CwaA (Buntin et al., 2017). In this study, 1 S-layer protein, 1 CnBP and 9 Mubs were annotated in W2 genome. These proteins may help W2 to adhere to mucus/intestinal epithelium and further research into this aspect is needed for validation.

*In vitro* antagonistic activity against pathogens is one of the more important aspects for the evaluation of beneficial properties of probiotics (Fijan et al., 2018; Doan et al., 2021). In this study, *L. plantarum* W2 showed strong antagonistic activity against seven common aquatic pathogenic bacteria, with inhibition zone diameters ranging from 13.6 to 27.3 mm. Typical antagonistic substances found in lactic acid bacteria include hydrogen peroxide (Bao et al., 2010), proteins and peptides (Selegard et al., 2019), organic acids (Sanchez-Maldonado et al., 2011; Arena et al., 2016), diacetyl and other compounds (Yan et al., 2019). The effective antibacterial substances of W2 existed in the supernatant and antagonistic activity was pH-dependent. Such pH-dependent antagonistic substances were also found in other strains of *L. plantarum*. Russo et al. (2017) demonstrated that the antagonistic ability of a *L. plantarum* strain against 6 filamentous fungi, including *Aspergillus niger*, derived from organic acids, with lactic acid playing an important role. Gerez et al. (2010) found that the antifungal activity of *L. plantarum* SL778 was associated with acetic acid, phenyllactic acid, and lactic acid. They also found that higher concentrations of lactic acid production favored the combined antagonistic activity of organic acids. In addition, the accumulation of antagonistic activity occurred when more than one organic acid was involved (Sun et al., 2003). Accordingly, it could be presumed that the antagonistic activity of W2 against the seven pathogens might be derived from organic acids. The exact components of these organic acids still need to be further analyzed in future study.

Until now, the *in vitro* efficacy and potency of many probiotics against numerous pathogens have been investigated under various conditions (Fijan et al., 2018; Doan et al., 2021). There are, however, many gaps and questions remaining to be clarified, especially the determination of the level of correlation of the *in vitro* antagonistic activity and the *in vivo* disease resistance of putative probiotics against pathogenic strains. Strain W2 was found to significantly increase the immune performance of shrimp, as evidenced by a general increase in serum immune enzyme activities and hepatopancreas immune-related gene expression levels. In the challenge test, W2 supplementation significantly enhanced *in vivo* pathogen resistance of shrimp under *V. parahaemolyticus* exposure. These results were in accordance with other studies. For example, Zheng et al. (2017) measured the expression levels of non-specific immune genes before and after acute hyposalinity stress in shrimp and showed that dietary supplementation with *L. plantarum* significantly improved the stress resistance and immune performance of shrimp. Dash et al. (2015) demonstrated that dietary inactivated *L. plantarum* could enhance non-specific immunity in *P. vannamei*. Chomwong et al. (2018) found that the use of *L. plantarum* promoted the immune response of shrimp to VP_*AHPND*_ infection and improved survival. Based on the above, *L. plantarum* W2 revealed better performance *in vivo* efficacy and potency of improving the immune capacity and disease resistance in shrimp.

In recent years, there has been increasing concern about the safety of probiotics due to the potential transfer and spread of ARGs and VFs among microorganisms (Wang et al., 2008; Doron and Snydman, 2015). A systematic safety assessment of *L. plantarum* W2 was conducted based on the latest EFSA guidelines for feed-added probiotics (EFSA, 2018) and other relevant studies (e.g., FAO/WHO, 2002; EFSA, 2008; WHO, 2014). The evaluation mainly focused on phenotypic and genotypic studies of antibiotic resistance and virulence, and to some extent on the biological and ecological risks of these genes. In this study, 274 VFs were found in the W2 genome although, fortunately, none was any of the genes that need to be excluded according to EFSA guidelines. The 274 VFs mainly perform auxiliary functions such as adhesion, anti-phagocytosis, ion uptake, secretion, and regulation. For pathogenic strains, adhesion and iron uptake are important for their colonization and invasion (Becker and Skaar, 2014; Carpenter and Payne, 2014). However, for probiotics, numerous studies have shown that adhesion to the intestinal surface is essential to its developing probiotic effects. *L. plantarum* is a kind of bacteria that does not need to absorb iron from its environment (Archibald, 2006). Therefore, some of the VFs might be important for probiotics to exert beneficial effects and should not be considered as malignant virulence factors.

There were 22 toxins in the 274 VFs, including 17 haemolysins/cytolysins, but strain W2 showed no hemolytic activity. These genes may not be expressed in W2, or weakly expressed. Other non-haemolytic toxins, including Colibactin, RTX toxin and TcdA, were difficult to identify as toxic to organisms if they were not highly expressed. Some studies have shown that the expression of some toxins, such as TcdA (Kuehne et al., 2010), can activate host innate immunity to some extent. VFs may be transferred from non-pathogenic bacteria to pathogens through MGEs-mediated Horizontal Gene Transfer (HGT) (von Wintersdorff et al., 2016; Brito, 2021). Gene mapping indicated that 10 VFs of W2 were located on GEIs and 6 were located near integrase/transposases, including no haemolysins/cytolysins genes. These genes may have some risk of HGT but have no predictable virulence to biology (Kuehne et al., 2010; Satchell, 2015).

Currently, antimicrobial resistance has been considered to be a global public health threat (WHO, 2014; CDC, 2019). W2 was considered as innately resistant to kanamycin, chloramphenicol, clindamycin, gentamicin, and tetracycline. However, the MICs of erythromycin and ampicillin against W2 were higher than their cut-off values (erythromycin: 1 μg/ml, ampicillin: 2 μg/ml). A new phenotype may signal a newly acquired gene. Gene mapping showed that none of the penicillin ARGs were located on GEIs/pre-phages or in the near vicinity of integrases and transposases. Thus, penicillin resistance of W2 might be innate resistance. Similarly, ampicillin resistance in *L. plantarum* was also described as innate resistance in other studies. For example, laboratory adaptive evolution experiments showed that chronic exposure to ampicillin up-regulated MIC of *L. plantarum* strain, but ampicillin ARGs transfer risk was low (Cao et al., 2020). Gene mapping showed that there are 4 macrolide efflux pump genes located on GEIs in W2. These genes might be acquired genes. HGT is a powerful motivator for bacterial evolution. It is not surprising that *L. plantarum*, a class of metabolically rich and adaptive bacteria, has acquired new genes from the environment. The increased resistance of W2 to erythromycin might derive from these genes in part, for which further evidence is still needed in future research.

Overall, a total of 11 ARGs, in which 7 are located on GEIs and 4 are located near integrases or transposases, were at some risk of gene transfer. However, on the one hand not all ARGs pose a severe threat to public health. Some genes or sequence homology predicted genes that have conferred antibiotic resistance are widespread in bacteria and play biological roles, such as efflux systems (Blair et al., 2015) and intercellular signaling (Davies and Davies, 2010). On the other hand, genes with transfer potential are not always prevalent on a large scale or have detrimental effects to humans. For example, a recent study by Zhang et al. (2021) suggested that the risk assigned to vanA and sul1 resistance families (resistant to vancomycin and sulforaphane, respectively) should be weakened. These two resistant families belong to the 37 ARG families of Rank I listed by WHO (2019). Studies have shown that they are characterized by gene transfer and host pathogenicity but show weak correlation with human activity. Because gene transfer is constrained by the influence of complex regulatory factors, there may be many unknown environmental and host factors (Wagner et al., 2017). Zhang et al. (2021) classified high-risk mobile ARGs into two classes: Rank I, containing 73 gene families that currently have a high risk of conferring new or multiple drug resistance to pathogen; Rank II, containing 19 gene families that may be transferred to pathogens in new forms of resistance in the future. In this study, none of the ARGs in W2 belonged to Rank I and 3 genes belonged to the mdtG family of Rank II, which conferred multi-drug resistance. However, the 3 genes were neither located on GEIs nor in the vicinity of integrases/transposases. Based the above, W2 may have high biological and ecological safety profiles, in terms of virulence and antimicrobial resistance.

Generally, *L. plantarum* is a common lactic acid bacterium with a long history of safe use and rarely leads to human infection (Bernardeau et al., 2006; Haghighat and Crum-Cianflone, 2016; Le and Yang, 2018; Nayeem et al., 2018; Koyama et al., 2019). As a potential probiotic strain for shrimp, W2 did not carry highly virulent factors by genome analysis, and its non-haemolytic activity was confirmed by haemolysis tests. Meanwhile, according to the omics-based framework for assessing the health risk of antimicrobial resistance genes (Zhang et al., 2021), W2 also has a low risk of facilitating the formation of multidrug resistant pathogens by conferring ARGs to pathogen both in the present and foreseeable future. Hence, based on the above collectively, it could be believed that W2 should have no or low potential risk to public health.

## Conclusion

The probiotic properties and safety of *L. plantarum* W2 were evaluated in this study. Strain W2 represented no significant virulence effect. W2 showed antibiotic resistance to ampicillin and erythromycin to a certain degree, i.e., inherently ampicillin resistant but might acquire resistance to erythromycin. However, it was confirmed that none of the ARGs in W2 belong to the high-risk mobile ARGs. Strain W2 has the ability to adhere to surfaces and can attenuate the adhesion of *V. parahaemolyticus*. Seven aquatic pathogenic strains were *in vitro* inhibited efficiently, and organic acids might be the main antagonistic substance produced by W2. Dietary W2 with 1 × 10^10^ cfu/kg live cells significantly improved the growth performance, enhanced levels of serological immune indicators and the expression levels of hepatopancreatic immune genes of shrimp and reduced the mortality of shrimp exposed to *V. parahaemolyticus*. Based on these results, *L. plantarum* W2 can be applied safely as a potential probiotic in shrimp farming to enhance growth performance, immunity capacity and disease resistance of *P. vannamei*. Additionally, the results from this study would be helpful to develop criteria for the screening and evaluation of probiotics in aquaculture.

## Data Availability Statement

The original contributions presented in the study are publicly available. This data can be found here: https://www.ncbi.nlm.nih.gov/bioproject/, PRJNA797524.

## Ethics Statement

All animal experiments were conducted in accordance with the guidelines and approval of the respective Animal Research and Ethics Committees of Ocean University of China (Permit Number: 20141201; http://www.gov.cn/gongbao/content/2011/content_1860757.htm).

## Author Contributions

CW performed the experiments, analyzed the data, and wrote the manuscript with assistance of KL, MW, YML, MP, YX, GQ, YJL, and LL. QL helped to collect and maintain the animals. XT provided the experimental ideas and the design of this study. All authors contributed to the article and approved the submitted version.

## Conflict of Interest

QL was employed by the Qingdao Ruizi Group Co. The remaining authors declare that the research was conducted in the absence of any commercial or financial relationships that could be construed as a potential conflict of interest.

## Publisher’s Note

All claims expressed in this article are solely those of the authors and do not necessarily represent those of their affiliated organizations, or those of the publisher, the editors and the reviewers. Any product that may be evaluated in this article, or claim that may be made by its manufacturer, is not guaranteed or endorsed by the publisher.
