# Supplementary material for: Evaluation of Potential Probiotic Properties of a Strain of Lactobacillus plantarum for Shrimp Farming: From Beneficial Functions to Safety Assessment
Source: Front Microbiol. 2022 Mar 24;13:854131. doi: 10.3389/fmicb.2022.854131 (PMC8989281; doi:10.3389/fmicb.2022.854131)
Supplement: Supplementary file 1 [file Data_Sheet_1.docx]

Supplementary Material

**Table S1** Nutritional compositions of basal diets

| **Ingredients** | **Content (%)** | **Ingredients** | **Content (%)** |
| --- | --- | --- | --- |
| Crude protein | ≥ 42.0 | Moisture | ≤ 12.0 |
| Crude fat | ≥ 4.0 | Lysine | ≥ 2.4 |
| Crude fiber | ≤ 3.0 | Total phosphorus | ≥ 1.0 |
| Crude ash | ≤ 15.0 |  |  |

**Table S2** Immune-related genes and primer sequences

| **Gene name** | **Gene description** | **Forward primers (5'-3')** | **Reverse primers (5'-3')** | **Source or accession number** |
| --- | --- | --- | --- | --- |
| *Actin* | Reference gene | GAGCAACACGGAGTTCGTTGT | CATCACCAACTGGGACGACATGGA | Sun et al., 2012 |
| *SOD* | Genes encoding superoxide dismutase that metabolise ROS (Campa-Córdova et al., 2002) | AGCCAATGACGTAAGCG | ACCATCACAAGAAACCC |  |
| *LZM* | Genes encoding lysozyme with antibacterial activity (Bergamo et al., 2019) | TGTTCCGATCTGATGTCC | GCTGTTGTAAGCCACCC |  |
| *proPO* | Genes encoding [prophenoloxidase](https://www.sciencedirect.com/topics/agricultural-and-biological-sciences/prophenoloxidase) which are associated with the formation of Melanization (Amparyup et al., 2013) | TCCATTCCGTCCGTCTG | GGCTTCGCTCTGGTTAGG |  |
| *LGBP* | Genes encoding lipopolysaccharide and beta-1,3-glucan binding protein, which are pathogen recognition receptors in crustaceans (Li and Xiang, 2013) | CATGTCCAACTTCGCTTTCAGA | ATCACCGCGTGGCATCTT | AY723297 |
| *HSP70* | Genes encoding heat shock protein 70, which act as an immune protective synergism (Zhou et al., 2010) | AACGATTCTCAGCGTCAGG | AGGTGCCACGGAACAGAT | AY645906 |
| *Imd* | Involved in Toll and Imd signaling pathways, responses to Gram-negative bacteria and leads to the activation of *Relish* (Liu et al., 2016) | TCACATTGGCCCCGTTATCC | ATCTCGCGACTGCACTTCAA | FJ592176 |
| *Toll* | Involved in Toll and Imd signaling pathways, regulates the expression of antimicrobial peptide genes (Liu et al., 2016) | TGGACTTCTGCTCGGACAAC | GTACATGTCCTTGGTCGGCA | DQ923424 |
| *Relish* | Genes encoding nuclear transcription factors Relish, which involved in humoral immunity (Huang et al., 2009) | CCTGTGAAGACATTAGGAGGAGTA | CCAGTTGTGGCATTCTTTAGG | Ge et al., 2014 |
| *TOR* | Involved in [mTOR signaling pathway](https://www.so.com/link?m=bZQOtgmpwS53gw4TIVQhA9j0LsiJAm0sOUCrpdpU%2Bi2qWNe9tUor6fbDIiuC%2BFjQCC9Hh1WtY148YBJKXM0UbxDi1ZUsd22izBOq5WsX8tOAU3rpG4xO%2B1i%2BKbeUQjUt4VeYTKTEHC%2FMbz57YrGma%2BU4NZOA%3D), which plays a crucial role in nutrition regulation (Jewell and Guan, 2013) | TGCCAACGGGTGGTAGA | GGGTGTTTGTGGACGGA | Duan et al., 2017 |
| *4E-BP* | Important regulator of overall translation levels in cells, controlled by TOR and acts on the cap-binding protein eIF4E (Teleman et al., 2005) | ATGTCTGCTTCGCCCGTCGCTCGCC | GGTTCTTGGGTGGGCTCTT |  |
| *eIF4E1α* | Genes encoding cap-binding protein eIF4E, which are closely associated with immunity (Piron et al., 2010) | TCCCTTTCCCTAACCCTCA | GTTTTGCTGTCTCGCTTCC |  |
| *eIF4E2* |  | TGGAATCAAACCTATGTGGG | GTCCTCCTGGAAGCGTA |  |

**Table S3** Physiological and biochemical characteristics of W2

| **Projects** | **W2** | **Projects** | **W2** |
| --- | --- | --- | --- |
| Gram | ＋ | Spores | － |
| Sucrose | ＋ | arabinose | ＋ |
| Fructose | ＋ | Mannitol | ＋ |
| Maltose | ＋ | Galactose | ＋ |
| D-ribose | ＋ | Xylose | － |
| L-rhamnose | ＋ | H_2_S | － |
| Lactose | ＋ | motility | － |
| Laetrile | ＋ | catalase | － |
| Trehalose | ＋ | MR | － |
| Mannose | ＋ | VP-test | ＋ |

Note:＋indicate positive results,－indicate negative results.

**Table S4** Classifications of antibiotic resistance genes of *L. plantarum*

| **Drug Class** | **Gene No.** |
| --- | --- |
| macrolide antibiotic | 50 |
| tetracycline antibiotic | 34 |
| fluoroquinolone antibiotic | 19 |
| phenicol antibiotic | 14 |
| lincosamide antibiotic | 14 |
| peptide antibiotic | 11 |
| penam | 11 |
| streptogramin antibiotic | 10 |
| oxazolidinone antibiotic | 10 |
| pleuromutilin antibiotic | 9 |
| aminocoumarin antibiotic | 9 |
| glycopeptide antibiotic | 8 |
| rifamycin antibiotic | 7 |
| acridine dye | 7 |
| nitroimidazole antibiotic | 7 |
| aminoglycoside antibiotic | 6 |
| cephalosporin | 6 |
| mupirocin | 4 |
| diaminopyrimidine antibiotic | 4 |
| carbapenem | 3 |
| cephamycin | 3 |
| fosfomycin | 3 |
| isoniazid | 2 |
| monobactam | 2 |
| sulfonamide antibiotic | 1 |
| fusidic acid | 1 |
| sulfone antibiotic | 1 |
| penem | 1 |
| triclosan | 1 |
| antibacterial free fatty acids | 1 |

**Table S5** Macrolides and penicillin resistance genes

1. Macrolides resistance genes in W2

| **Database** | **Resistance mechanism** | **Number** | **ARO Name** |
| --- | --- | --- | --- |
| CARD | antibiotic effluxs | 42 | 3efrA, 2evgA, 25macB, MexL, 3mtrA, 5oleC, 3Staphylococcus aureus LmrS |
|  | antibiotic target protection | 7 | lmrC, lsaA, lsaC, 2optrA, tva(A), vmlR |
|  | antibiotic target alteration | 1 | Erm(K) |
| Count | | 50 |  |

1. Penicillin resistance genes in W2

| **Database** | **Resistance mechanism** | **Number** | **ARO Name/Gene site** |
| --- | --- | --- | --- |
| CARD | antibiotic effluxs | 7 | 3mtrA,mgrA,2evgA,golS |
|  | antibiotic target protection | 1 | mecI |
|  | antibiotic inactivation | 3 | Escherichia coli ampH beta-lactamase,y56 beta-lactamase,NmcR |
| Swiss-Prot | antibiotic target (penicillin-binding protein) | 5 | gene0966 gene1180 gene1318 gene1480 gene1906 |
| pfam | antibiotic inactivation (β-lactamase) | 13 | gene0037，gene0410，gene0966，gene1059，gene1708，gene1815，gene1844，gene1850，gene1874，gene2056，gene2093，gene2774，gene2882 |
| NR | penicillin V acylase | 1 | gene0056 |
| GO | penicillin amidase activity | 1 | gene2921 |
| Count | | 27 |  |

**Table S6** Genes site of 22 toxin

| **Toxin** | **Number** | **Gene site** |
| --- | --- | --- |
| alpha-Hemolysin (VF0225) | 4 | gene0254，gene0368，gene1471，gene2371 |
| Beta-hemolysin/cytolysin (CVF171) | 11 | gene0406，gene1411，gene1616，gene1658 gene2201，gene2398，gene2464，gene2490 gene2601，gene2696，gene3155 |
| Colibactin (VF0573) | 1 | gene1652 |
| Cytolysin (VF0356) | 1 | gene2524 |
| Hemolysin III (CVF793) | 1 | gene2859 |
| RTX toxin (CVF263) | 3 | gene0876，gene1048，gene1389 |
| TcdA (VF0376) | 1 | gene3123 |

**References**

Amparyup, P., Charoensapsri, W., Tassanakajon, A. (2013). Prophenoloxidase system and its role in shrimp immune responses against major pathogens. *Fish Shellfish Immunol.* 34: 4, 990-1001. doi: 10.1016/j.fsi.2012.08.019

Bergamo, A., Gerdol, M., Pallavicini, A., Greco, S., Schepens, I., Hamelin, R., et al. (2019). Lysozyme-Induced Transcriptional Regulation of TNF-α Pathway Genes in Cells of the Monocyte Lineage. *Int J Mol Sci* 20: 21, 5502. doi: 10.3390/ijms20215502

Campa-Córdova, A. I., Hernández-Saavedra, N. Y., Ascencio, F. (2002). Superoxide dismutase as modulator of immune function in American white shrimp (*Litopenaeus vannamei*). *Comp Biochem Physiol C Toxicol Pharmacol* 133: 4, 557-565. doi: 10.1016/S1532-0456(02)00125-4

Duan, Y., Zhang, Y., Dong, H., Wang, Y., Zhang, J., (2017). Effects of dietary poly-β-hydroxybutyrate (PHB) on microbiota composition and the mTOR signaling pathway in the intestines of *litopenaeus vannamei*. *J. Microbiol.* 55, 946–954. doi: 10.1007/s12275-017-7273-y.

Ge, Q.Q., Li, J., Liang, J.P., Liu, P., Zhao, F.Z., Pan, L.Q., et al. (2014). Cloning of Imd immune signal pathway relating genes of *fenneropenaeus chinensis* and their expression analysis. Period. *Ocean University of China* 44, 46–51

Huang, X. D., Yin, Z. X., Liao, J. X., Wang, P. H., Yang, L. S., Ai, H. S., et al. (2009). Identification and functional study of a shrimp Relish homologue. *Fish Shellfish Immunol.* 27: 2, 230-238. doi: 10.1016/j.fsi.2009.05.003

Jewell, J. L., Guan, K.-L. (2013). Nutrient signaling to mTOR and cell growth. *Trends Biochem. Sci.* 38: 5, 233-242. doi: 10.1016/j.tibs.2013.01.004

Li, F., Xiang, J. (2013). Recent advances in researches on the innate immunity of shrimp in China. *Dev. Comp. Immunol.* 39: 1, 11-26. doi: 10.1016/j.dci.2012.03.016

Liu, Y., Song, L., Sun, Y., Liu, T., Hou, F., Liu, X. (2016). Comparison of immune response in Pacific white shrimp, *Litopenaeus vannamei*, after knock down of Toll and IMD gene in vivo. *Dev. Comp. Immunol.* 60, 41-52. doi: 10.1016/j.dci.2016.02.004

Piron, F., Nicolaï, M., Minoïa, S., Piednoir, E., Moretti, A., Salgues, A., et al. (2010). An induced mutation in tomato eIF4E leads to immunity to two potyviruses. *PLoS One* 5: 6, e11313. doi: 10.1371/journal.pone.0011313

Sun, Y., Liu, F., Song, X., Mai, K., Li, Y., Huang, J., (2012). Effects of adding probiotics in the feed on non-specific immune gene expression and disease resistance of *Litopenaeus vannamei*. *Oceanologia et Limnologia Sinica* 43, 845–851. doi: 10.1007/s11783-011-0280-z.

Teleman, A. A., Chen, Y. W., Cohen S. M., (2005). 4E-BP functions as a metabolic brake used under stress conditions but not during normal growth. *Genes Dev* 19(16), 1844-1848. doi: 10.1101/gad.341505

Zhou, J., Wang, W.-N., He, W.-Y., Zheng, Y., Wang, L., Xin, Y., et al. (2010). Expression of HSP60 and HSP70 in white shrimp, *Litopenaeus vannamei* in response to bacterial challenge. *J Invertebr Pathol* 103: 3, 170-178. doi: 10.1016/j.jip.2009.12.006
